# Serum 1,25-Dihydroxyvitamin D: An Outcome Prognosticator in Human Sepsis

**DOI:** 10.1371/journal.pone.0064348

**Published:** 2013-05-31

**Authors:** H. Bryant Nguyen, Blen Eshete, K. H. William Lau, Adarsh Sai, Mark Villarin, David Baylink

**Affiliations:** 1 Department of Medicine, Loma Linda University, Loma Linda, California, United States of America; 2 Department of Emergency Medicine, Loma Linda University, Loma Linda, California, United States of America; 3 Musculoskeletal Disease Center, Jerry L. Pettis Memorial VA Medical Center, Loma Linda, California, United States of America; University of Cincinnati, United States of America

## Abstract

In sepsis, the vitamin D active metabolite 1,25-dihydroxyvitamin D (1,25(OH)_2_D) may play a crucial role by its action to produce cathelicidin and improve endothelial barrier function, such that a deficiency in 1,25(OH)_2_D is associated with poor outcome. To test our hypothesis, we performed analysis of stored plasma samples from a prospective observational study in 91 patients with sepsis, age of 59.1+/−2.0 years, 52.7% females, and 11.0% deaths at 30 days. Vitamin D status, including 25-hydroxyvitamin D (25(OH)D), 1,25(OH)_2_D, 24,25-dihydroxyvitamin D (24,25(OH)_2_D), and parathyroid hormone (PTH), were measured daily over 3 days after hospital admission. At baseline, 1,25(OH)_2_D was significantly different between survivors vs. non-survivors. But there was no significant difference in 25(OH)D, 24,25(OH)_2_D, and PTH. In a multivariable binomial logistic regression model, age, total calcium and 1,25(OH)_2_D were significant predictors of 30-day mortality. Kaplan Meier analysis showed that patients with mean 1,25(OH)_2_D measured over 3 days of < = 13.6 pg/mL had 57.1% 30-day survival compared to 91.7% in patients with 1,25 (OH)_2_D level >13.6 pg/mL (p<0.01). From repeated measures regression analysis, there was significant increase in 1,25(OH)_2_D for increases in 25(OH)D in both survivors and non-survivors. However, compared to survivors, the low 25(OH)D in non-survivors was insufficient to account for the larger decrease in 1,25(OH)_2_D, indicating a dysfunctional 1α-hydroxylase. Additionally, there was a significant negative correlation between PTH and 1,25(OH)_2_D in both survivors and non-survivors, suggesting a severe impairment in the effect of PTH to increase renal 1α-hydroxylase activity. In conclusion, low 1,25(OH)_2_D levels are associated with increased 30-day mortality in sepsis patients, likely due to impaired 25(OH)D hydroxylation and PTH insensitivity. Our data also suggest that the active metabolite 1,25(OH)_2_D may be an important therapeutic target in the design of sepsis clinical trials.

## Introduction

Decades of research in sepsis have explored various novel therapies targeting the inflammatory consequences of infection, including corticosteroids, anti-endotoxin antibodies, anti-tumor necrosis factor monoclonal antibody, interleukin-1 receptor antagonist, and a number of other anti-inflammatory molecules [1]. However, the common scenario of promising experimental observations had all followed with failed clinical trials. With the recent withdrawal of drotrecogin alfa (activated) from the worldwide market, based on results of the PROWESS-SHOCK study, and the failed phase III trial examining eritoran tetrasodium targeting toll-like receptor-4 inhibition, new insight are needed with respect to the immune regulation and pathogenic mechanisms involved in sepsis [2], [3].

Approximately 1 billion people worldwide have vitamin D *insufficiency* and/or *deficiency*, defined as 25-hydroxyvitamin D (25(OH)D) level<30 ng/mL or<20 ng/mL, respectively [4]. Critically ill patients with vitamin D deficiency have increased odds ratio for in-hospital mortality [5], [6]. Sepsis patients presenting to the emergency department (ED) and in the intensive care unit (ICU) have been shown to have lower levels of 25(OH)D compared to healthy controls [7], [8]. However, debate exists as to whether vitamin D deficiency is associated with mortality in sepsis [9]. Another variable that has not been well studied is the fact that 25(OH)D, the circulating form of vitamin D, is inactive and requires conversion to the active hormone, 1,25-dihydroxyvitamin D (1,25(OH)_2_D), by a renal (endocrine) and extra-renal (autocrine/paracrine) 1α-hydroxylase enzyme. The primary functions of the active 1,25(OH)_2_D is then mediated by its binding to the nuclear vitamin D receptor, resulting in neutrophil synthesis of antimicrobial peptides, cathelicidin and defensin [10], [11]. In cardiovascular disease, both 25(OH)D deficiency and inadequate 25(OH)D hydroxylation may result in severely deficient 1,25(OH)_2_D, such that 1,25(OH)_2_D itself is independently associated with mortality [12].

In this study, we aimed to examine the association of patient outcome to vitamin D status measured at 0, 24, 48 and 72 hours in patients with sepsis presenting to the emergency department and admitted to the hospital. We defined *vitamin D status* to include measurements of 25(OH)D; 1,25(OH)_2_D; 24,25-dihydroxyvitamin D (24,25(OH)_2_D)); and parathyroid hormone (PTH). We hypothesized that the active metabolite of vitamin D, 1,25(OH)_2_D, rather than 25(OH)D, is a prognosticator of 30-day mortality in sepsis.

## Materials and Methods

### Design and Setting

This study was a single-center analysis of stored plasma samples from subjects previously enrolled at our tertiary care institution for a prospective, multi-center, observational study of patients presenting to the ED with suspected sepsis and admitted to the medical ICU. A convenient sample of patients was enrolled from January 2005 through June 2006 for the study purpose of identifying biomarkers associated with poor patient outcomes. The results of the multi-center study have been published [13]. Our single-center analysis of stored samples for the current study was performed from September 2011 through April 2012. The study was approved by our Institutional Review Board for Human Research, and study subjects were enrolled with written informed consent.

### Patient Selection

Patients with age 18 years or older were considered for enrollment if they met the accepted definition for *sepsis*: 1) confirmed or suspected source of infection determined by the treating clinician and/or a serum lactate level >2.5 mmol/L; and 2) two or more criteria for the systemic inflammatory response syndrome (SIRS), defined as a) temperature greater than 38°C or less than 36°C; b) respiratory rate greater than 20 breaths per minute or partial pressure of carbon dioxide less than 32 mm Hg; c) heart rate greater than 90 beats per minute; and d) white blood cell count greater than 12,000 or less than 4,000 cells per mm^3^, or greater than 10% immature forms [14]. Patients were excluded if they experienced cardiac arrest on arrival, under a do-not-attempt-resuscitation order, or were pregnant.

### Data Collection

Physiologic data were obtained at enrollment, including demographics, comorbidities, Acute Physiology and Chronic Health Evaluation (APACHE) II scores, laboratories including cultures, and the suspected source of infection. *Septic shock* was defined as sepsis and the presence of hypotension (mean arterial pressure<70 mm Hg or systolic blood pressure<90 mm Hg) despite adequate fluid resuscitation or requiring vasopressor therapy [14]. We performed serial measurements at hour 0, 24, 48, 72, relevant to the vitamin D status. We also recorded 30-day mortality as the primary outcome measurement.

### Plasma Sample Collection and Vitamin D Status Measurements

After obtaining written informed consent, whole blood was collected at hour 0, 24, 48, and 72 after enrollment by venipuncture into collection tubes containing ethylenediaminetetraacetic acid as an anticoagulant. Within 1 hour of collection, each sample was centrifuged at 2,000×g for 10 minutes. The plasma was immediately aliquoted into 1 mL cryovials and stored at −84°C without further freeze-thaw cycles. For our study purposes, the samples (which included 1 mL at each time point) were subsequently shipped in dry ice to Heartland Assay, LLC, Ames, Iowa, for analysis of 25-hydroxyvitamin D (25(OH)D); 1,25-dihydroxyvitamin D (1,25(OH)_2_D); 24,25-dihydroxyvitamin D (24,25(OH)_2_D); and parathyroid hormone (PTH).

#### 25-hydroxyvitamin D (25(OH)D)

The method for quantitative determination of 25(OH)D is an FDA approved direct, competitive chemiluminescence immunoassay (CLIA) using the DiaSorin LIAISON 25-OH Vitamin D Total assay [15], [16]. This assay is co-specific for 25(OH)D_3_ and 25(OH)D_2_. The assay utilizes a specific antibody to 25(OH)D coating magnetic particles (solid phase) and a vitamin D analogue, 22-carboxy-23,24,25,26,27-pentanorvitamin D_3_, linked to an isoluminol derivative. During the incubation, 25(OH)D is dissociated from its binding protein, and competes with the isoluminol labeled analogue for binding sites on the antibody. After the incubation, the unbound material is removed with a wash cycle. Subsequently, the starter reagents are added and a flash chemiluminescent reaction is initiated. The light signal is measured by a photomultiplier as relative light units (RLU) and is inversely proportional to the concentration of 25(OH)D present in calibrators, controls, or samples. The assay has a normal range of 30 to 80 ng/mL, with inter- and intra-assay coefficients of variability of 11.2% and 8.1%, respectively.

#### 1,25-dihydroxyvitamin D (1,25(OH)2D)

The assay for 1,25(OH)_2_D involves a preliminary extraction and subsequent purification of vitamin D metabolites from serum or plasma using C_18_OH cartridges. Following extraction, the treated sample is then assayed using a competitive RIA procedure [17]. The RIA method is based on a polyclonal antibody that is specific for both 1,25(OH)_2_D_2_ and 1,25(OH)_2_D_3_. The sample, antibody and tracer are incubated for 2 hours at 20–25°C. Phase separation is accomplished after 20-minute incubation at 20–25°C with a second antibody precipitating complex. After centrifugation and decantation, the bound fraction remaining in the pellet is counted in a gamma counter. Values are calculated directly from a calibrator curve of known concentrations. The assay has a normal range of 20 to 50 pg/mL, with inter- and intra-assay coefficients of variability of 12.6% and 9.8%, respectively.

#### 24,25-Dihyoxyvitamin D (24,25(OH)2D)

The 24,25(OH)_2_D level was measured by a modification of the procedure described by Horst et al. [18]. Briefly, plasma lipids were extracted and purified by silica solid phase extraction (SPE) and finally by high pressure liquid chromatography (HPLC). The HPLC purified material which contained both 24,25(OH)_2_D_2_ and 24,25(OH)_2_D_3_ was assayed by radioimmunoassay (RIA). [^3^H]-24,25(OH)_2_D_3_ was used to estimate losses. The RIA method was based on an antibody which was co-specific for 24,25(OH)_2_D_2_ and 24,25(OH)_2_D_3_ [19]. The sample, antibody and tracer are incubated for 120 minutes at 20–25°C. Phase separation is accomplished after 20-minute incubation at 20–25°C with a second antibody precipitating complex. A NSB/Addition buffer is added after this incubation prior to centrifugation to aid in reducing non-specific binding. Radioactivity was quantitated by the γ-radiation counting system with use of a smooth-spline method. The assay has a normal range of 1 to 3 ng/mL, with inter- and intra-assay coefficients of variability of 10.0% and 8.0%, respectively.

#### Parathyroid hormone (PTH)

PTH assays were performed using the FDA approved DiaSorin intact PTH immunoradiometric assay (IRMA). This assay utilizes two different polyclonal antibodies that have been purified using affinity chromatography. These purified antibodies are specific for two different regions of the PTH molecule. The first antibody, specific for PTH 39–84 is bound to a solid phase (polystyrene bead). The second antibody is specific for PTH 1–34 and is labeled with iodine-125. Samples are incubated simultaneously with both antibodies, intact PTH 1–84 contains both the 1–34 and 39–84 amino acid sequences and is the only form of PTH that will be bound to both the antibody on the bead and the antibody labeled with iodine-125. Since the antibody coupled to the solid phase is specific for C-terminal and mid-region fragments as well as intact PTH, the capacity of the solid phase has been designed to accommodate very high levels of PTH. This prevents interference by extremely elevated C-terminal and mid-region PTH fragments in unknown samples. Following the incubation period, each bead is washed to remove any unbound labeled antibody. The radioactivity present in the remaining bound labeled antibody is then measured using a gamma counter. Concentrations of intact PTH present in the samples are directly proportional to the radioactivity measured. The assay has a normal range of 15 to 65 pg/mL, with inter- and intra-assay coefficients of variability of 4.3% and 2.7%, respectively.

### Statistical Analysis

Baseline characteristics of the study population were compared between survivors and non-survivors using student’s T-test for normally distributed continuous variables, and Mann-Whitney U test for skewed variables. Chi-square was used for categorical variables (Table 1).

**Table 1:** Patient characteristics in survivors and non-survivors.

	All Patients (N = 91)	Survivors (N = 81)	Non-survivors (N = 10)	*p-value*
Age, years	59.1±2.0	57.6±2.2	71.4±3.1	*0.03*
Male:Female	43∶48	39∶42	4∶6	*0.63*
Racial background				*0.55*
Causian	57	52	5	
Hispanic	18	16	2	
Asian	12	9	3	
Black	2	2	0	
Others	1	1	0	
Height, m	1.67±0.01	1.67±0.01	1.67±0.02	*0.91*
Weight, kg	75.0±2.0	75.3±2.3	71.5±4.8	*0.58*
Body mass index	27.2±0.9	27.4±1.1	25.85±1.9	*0.58*
Comorbidities, no (%)				
Hypertension	38 (41.8)	33 (40.7)	5 (50.0)	*0.64*
Diabetes	34 (37.4)	30 (37.0)	4 (40.0)	*0.88*
Coronary disease	29 (31.9)	24 (29.6)	5 (50.0)	*0.22*
Stroke	13 (14.3)	9 (11.1)	4 (40.0)	*0.02*
Cirrhosis	2 (2.2)	1 (1.2)	1 (10.0)	*0.07*
Kidney disease	6 (6.6)	4 (4.9)	2 (20.0)	*0.08*
Chronic hemodialysis	3 (3.3)	3 (3.7)	0 (0.0)	*0.55*
Thromboembolism	2 (2.2)	2 (2.5)	0 (0.0)	*0.63*
Smoking	14 (15.4)	12 (14.8)	2 (20.0)	*0.60*
Alcohol dependency	13 (14.3)	11 (13.6)	2 (20.0)	*0.53*
Recreational drugs	8 (8.8)	7 (8.6)	1 (10.0)	*0.83*
Immunocompromised	6 (6.6)	6 (7.4)	0 (0.0)	*0.39*
Physiologic Score				
APACHE II	21.5±1.9	19.2±1.7	37.0±4.5	*<0.01*
APACHE II Predicted mortality, %	41.7±5.0	36.0±4.7	81.7±8.4	*<0.01*
Septic shock, no (%)	9 (9.9)	5 (6.2)	4 (40.0)	*0.01*
Blood culture positive, no (%)	24 (26.4)	20 (24.7)	4 (40.0)	*0.31*
Culture positive, no (%)	59 (64.8)	50 (61.7)	9 (90.0)	*0.08*
Laboratories				
White blood cell, per mm^3^	17.3±1.4	17.5±1.6	16.0±3.6	*0.76*
Percent bands, %	27.1±3.5	26.3±3.6	34.3±13.5	*0.51*
Hematocrit, %	35.6±0.7	35.9±0.7	33.3±2.3	*0.25*
Platelet, 10^3^/µL	287.7±17.1	284.0±17.3	317.3±70.3	*0.54*
Glucose, mg/dL	142.8±6.3	139.2±6.4	175.3±24.2	*0.08*
Lactate, mmol/L	3.2±0.3	3.1±0.3	3.7±0.9	*0.51*
Source of Infection, no (%)				
Pneumonia	31 (34.1)	25 (30.9)	6 (60.0)	*0.07*
Urinary tract	24 (26.4)	23 (28.4)	1 (10.0)	*0.21*
Intra-abdominal	8 (8.8)	6 (7.4)	2 (20.0)	*0.19*
Soft tissue	2 (2.2)	2 (2.5)	0 (0.0)	*0.62*
Catheter related	5 (5.5)	4 (4.9)	1 (10.0)	*0.52*
Others	25 (27.5)	24 (29.6)	1 (10.0)	*0.19*

Data are presented as *mean ± standard error*, or *count (percentage of column).* APACHE – Acute Physiology and Chronic Health Evaluation.

A *Time* variable was created to represent repeated measurements over hour 0, 24, 48, and 72. This variable was utilized in repeated measures analysis of variance (ANOVA), which was performed on the clinically important variables related to the vitamin D axis to test for the significance of changes in values over time and to obtain least square estimated group means. Natural log transformations were required for variables that violate the assumptions of linear regression (Table 2).

**Table 2:** Vitamin D status and related variables measured over 72 hours that may be associated with 30-day mortality.

	Estimated mean	Hour 0	Hour 24	Hour 48	Hour 72	*Time trend* *p-value*	*Normal range*
25(OH)D, ng/mL	17.7±0.6*^†^*	21.5±1.1	18.0±1.2	22.0±1.6	19.0±1.4	*<0.01*	30 to 80 ng/mL
Survivors	18.2±0.7*^†^*	21.4±1.2	18.6±1.2	23.1±1.8	19.7±1.5	*0.14*	
Non-Survivors	16.9±1.3*^†^*	21.9±3.5	13.7±3.6	15.2±2.3	15.1±2.7	*0.24*	
* p-value*	*0.03*	*0.90*	*0.18*	*0.11*	*0.25*	*−*	
1,25(OH)_2_D, pg/mL	27.5±0.9*^†^*	30.5±1.6	29.4±2.0	33.0±2.9	32.3±2.9	*<0.01*	20 to 50 pg/mL
Survivors	29.4±0.9*^†^*	31.5±1.7	31.1±2.1	35.6±3.2	35.0±3.1	*0.44*	
Non-survivors	16.4±1.3*^†^*	20.4±3.4	17.0±2.5	15.8±1.3	16.2±1.4	*0.52*	
* p-value*	*<0.01*	*0.04*	*0.02*	*0.02*	*0.02*	*−*	
24,25(OH)_2_D, ng/mL	1.6±0.1	1.3±0.1	2.3±0.1	1.5±0.1	1.5±0.1	*<0.01*	1 to 3 ng/mL
Survivors	1.7±0.1	1.3±0.1	2.4±0.1	1.5±0.1	1.5±0.2	*<0.01*	
Non-survivors	1.5±0.2	0.9±0.1	2.2±0.3	1.4±0.3	1.5±0.4	*0.01*	
* p-value*	*0.21*	*0.14*	*0.40*	*0.62*	*0.88*	*−*	
PTH, pg/mL	57.7±3.1*^†^*	67.5±7.5	105.4±15.4	102.5±17.5	79.9±14.3	*<0.01*	15 to 65 pg/mL
Survivors	53.5±3.0*^†^*	65.1±7.8	92.1±14.7	88.3±16.7	66.8±9.3	*0.27*	
Non-survivors	98.1±13.1*^†^*	85.0±26.4	206.1±61.2	197.6±68.1	159.0±81.5	*0.39*	
* p-value*	*<0.01*	*0.40*	*0.01*	*0.03*	*0.02*	*−*	
Creatinine, mg/dL	1.5±0.1	1.6±0.1	1.5±0.2	1.3±0.1	1.6±0.2	*<0.01*	0.7 to 1.3 mg/dL
Survivors	1.5±0.1	1.5±0.2	1.4±0.3	1.2±0.2	1.6±0.3	*0.66*	
Non-survivors	1.7±0.3	1.6±0.3	2.3±0.4	1.5±0.4	1.5±0.4	*0.34*	
* p-value*	*0.40*	*0.89*	*0.17*	*0.55*	*0.91*	*−*	
Total calcium, mg/dL	8.2±0.1	8.9±0.1	7.5±0.2	7.7±0.2	8.1±0.2	*<0.01*	8.5 to 10.2 mg/dL
Survivors	8.3±0.1	8.9±0.1	7.6±0.3	7.9±0.2	8.3±0.2	*<0.01*	
Non-survivors	7.1±0.3	8.3±0.5	6.1±0.6	6.2±0.6	6.8±0.6	*0.02*	
* p-value*	*<0.01*	*0.10*	0.04	0.01	0.02	*−*	
Albumin, g/dL	3.0±0.1	3.2±0.1	2.6±0.2	2.4±0.1	2.5±0.2	*<0.01*	*3.8 to 5.2 g/dL*
Survivors	3.1±0.1	3.3±0.1	2.7±0.2	2.5±0.1	2.6±0.2	*<0.01*	
Non-survivors	2.2±0.2	2.5±0.3	1.7±0.3	1.8±0.3	2.2±0.0	*0.56*	
* p-value*	*<0.01*	*<0.01*	*0.07*	*0.07*	*0.31*	*−*	
Total bilirubin, mg/dL	1.2±0.1	1.2±0.1	1.1±0.2	1.1±0.1	0.9±0.2	*0.08*	0.3 to 1.9 mg/dL
Survivors	1.2±0.1	1.2±0.1	1.1±0.2	1.2±0.2	0.9±0.2	*0.50*	
Non-survivors	1.1±0.2	1.2±0.3	0.8±0.1	0.8±0.1	2.0±0.0	*0.70*	
* p-value*	*0.80*	*0.83*	*0.43*	*0.60*	*0.18*	*−*	

*Time trend p-value* describes the change in the variable over the four measurement periods derived from a repeated measure ANOVA. Overall *estimated mean* is the least-square mean derived from a repeated measure ANOVA. *^†^R*esults reflect analysis of the natural log transformed variables. Data are presented as *mean ± standard error*. 25(OH)D –25-dihydroxyvitamin D; 1,25(OH)_2_D –1,25-dihydroxyvitamin D; 24,25(OH)_2_D –24,25-dihydroxyvitamin D; PTH – parathyroid hormone.

Univariate analysis was performed on variables that were associated with 30-day mortality as the outcome variable. To obtain odds ratios, analysis of longitudinal data (Generalized Estimating Equations method - GEE) was then performed only on those variables that were significant in the univariate analysis. The final multivariable model was derived by forcing age into the model to test the main effect of 1,25(OH)_2_D on outcome, and adding one at a time, those variables in the univariate analysis that were significantly associated with the outcome variable. Significance as well as goodness-of-fit statistics was considered when selecting the appropriate final model. Transformation was not applied since GEE method of analysis is robust against assumption of homogeneity of variance (Table 3).

**Table 3:** Generalized estimating equations modeling using univariately statistically significant variables associated with 30-day mortality to derive a multivariable mortality prediction model.

	OR (95% CI)	*p-value*
**Univariate analysis**		
Age	1.04 (1.02, 1.07)	*<0.01*
Stroke comorbidity	5.11 (2.48, 10.52)	*<0.01*
APACHE II	1.20 (1.12, 1.25)	*<0.01*
Septic shock	30.54 (3.34, 279.42)	*<0.01*
Total calcium	0.60 (0.46, 0.81)	*<0.01*
Albumin	0.24 (0.10, 0.60)	*<0.01*
25(OH)D	0.95 (0.91, 1.00)	*0.06*
1,25(OH)_2_D	0.86 (0.81, 0.91)	*<0.01*
PTH	1.00 (1.00, 1.01)	*<0.01*
**Multivariable model**		
Age	1.08 (1.03, 1.12)	*<0.01*
Total calcium	0.55 (0.38, 0.80)	*<0.01*
1,25 (OH)_2_D	0.84 (0.76, 0.92)	*<0.01*

A *Time* variable reflecting repeated measurements over hour 0, 24, 48, and 72, was included and did not affect the results of the model at α = 0.05. Only 9 patients in our study had septic shock, resulting in a large 95% confidence interval for *septic shock* as a predictor of mortality. APACHE – Acute Physiology and Chronic Health Evaluation; 25(OH)D –25-dihydroxyvitamin D; 1,25(OH)_2_D –1,25-dihydroxyvitamin D; PTH – Parathyroid hormone; OR – odds ratio; CI – confidence interval.

**Figure 1:**
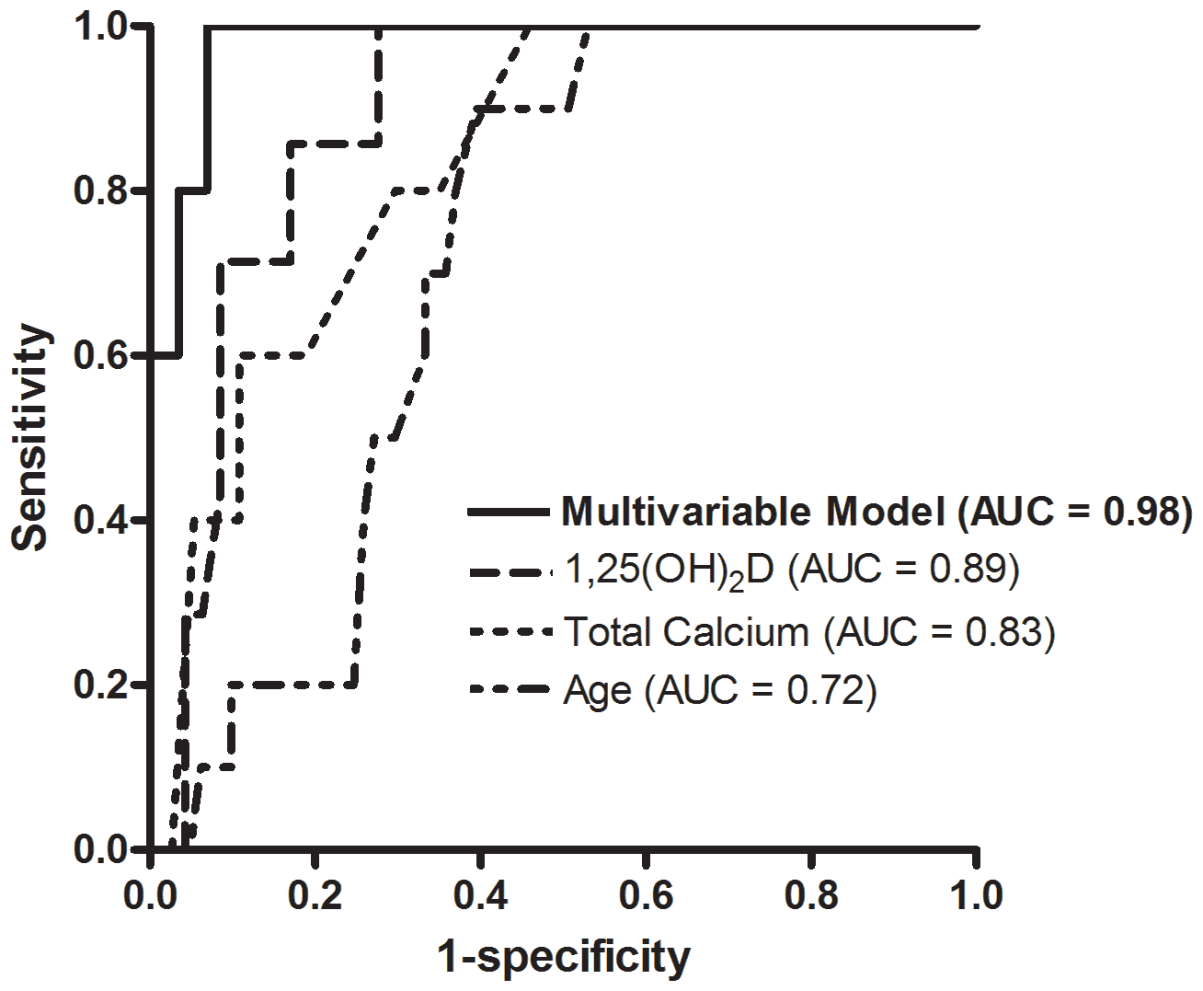
Receiver operating characteristic (ROC) curves for age, total calcium, 1,25-dihydroxyvitamin D (1,25(OH)_2_D) at hour 48, and the multivariable model including these 3 variables in discriminating survivors from non-survivors at 30 days after enrollment. AUC – Area under the ROC curve.

**Figure 2:**
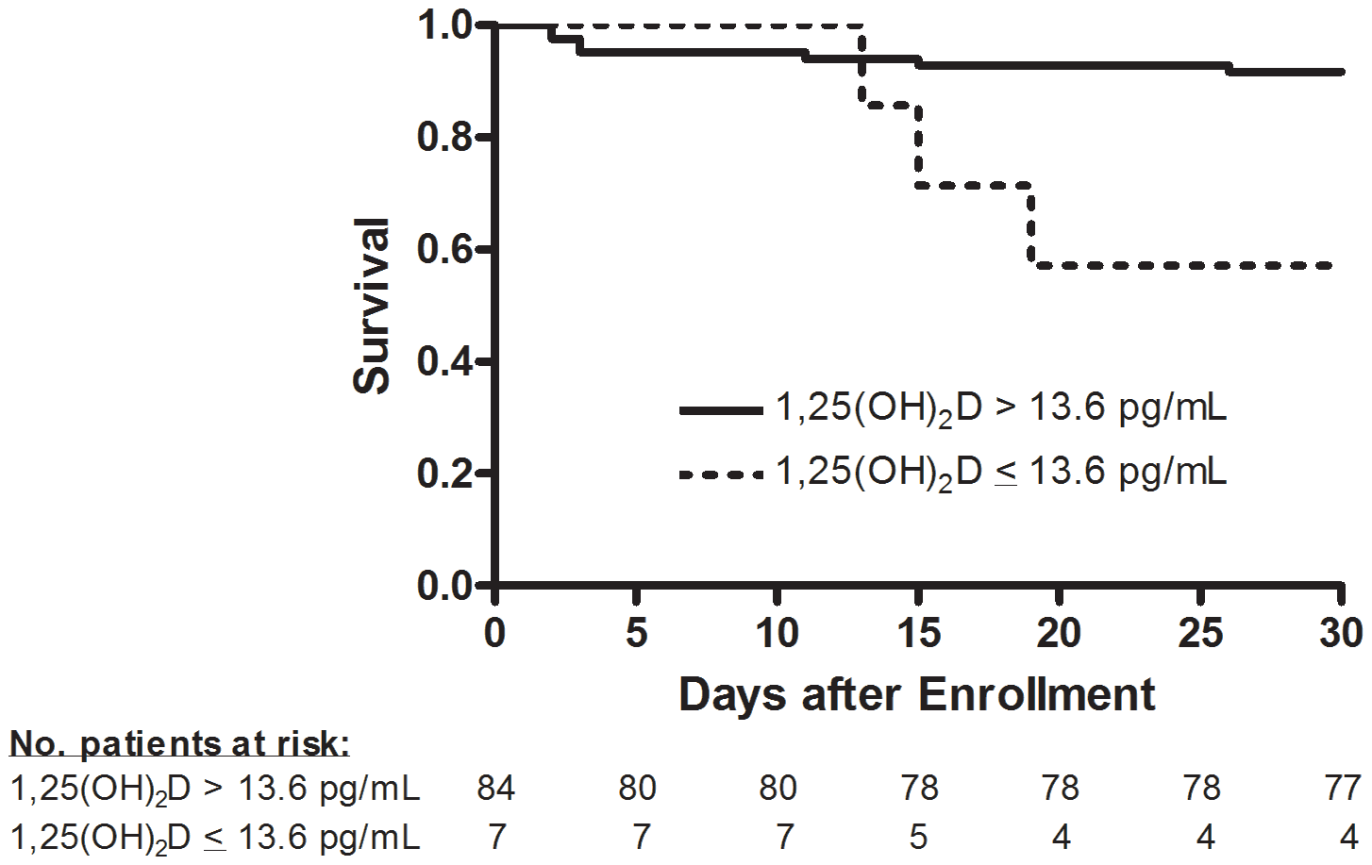
Kaplan Meier survival analysis between patients with mean 1,25-dihydroxyvitamin D (1,25(OH)_2_D)<vs.>13.6 pg/mL measured over the 72-study period. The mean survival time for patients with 1,25(OH)_2_D<13.6 pg/mL was 17.6 days, compared to 24.6 days in patients with 1,25 (OH)_2_D level >13.6 pg/mL (p<0.01).

Receiver operating characteristics (ROC) curves were generated to determine the ability of each significant variable in the model and the overall model to discriminate 30-day mortality (Figure 1). Kaplan-Meir analysis was then performed to obtain 30-day survival curves for the variable with the highest area under the ROC curve in the model (Figure 2).

**Figure 3:**
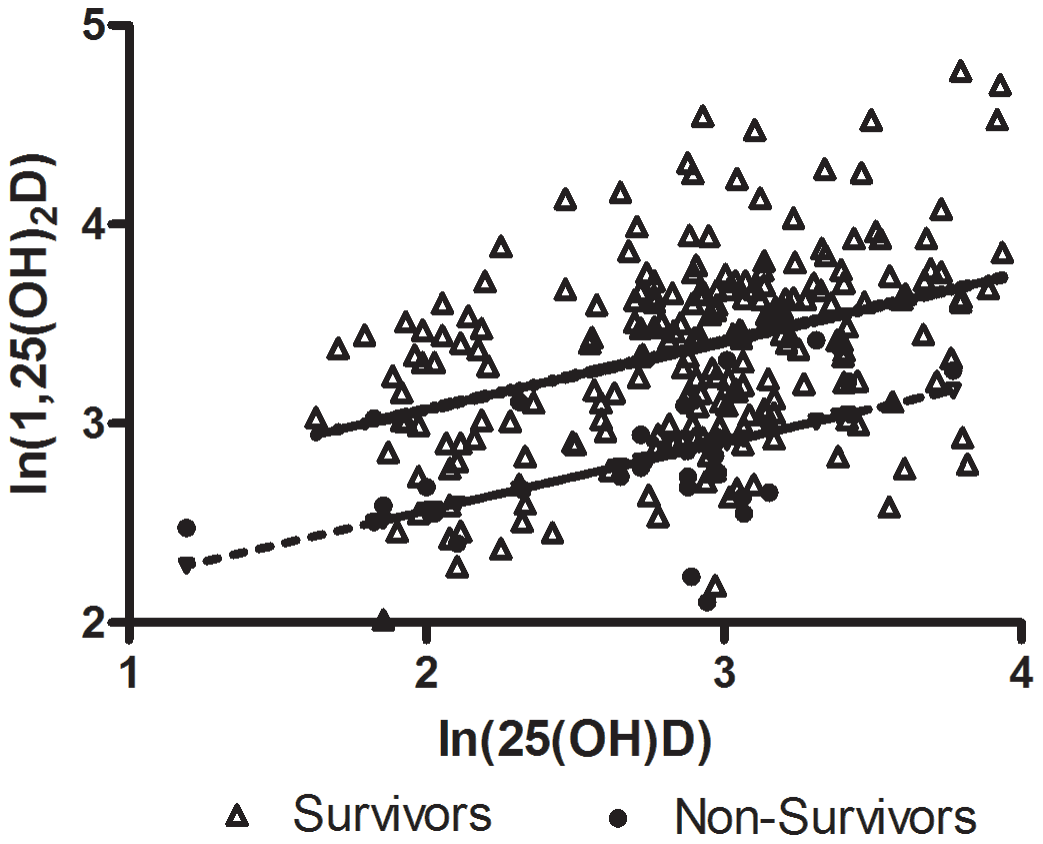
1,25-dihydroxyvitamin D (1,25(OH)_2_D) as a function of 25-hydroxyvitamin D (25(OH)D). In both survivors and non-survivors, there was a significant increase in ln(1,25(OH)_2_D) for increases in ln(25(OH)D) (p<0.01 and p = 0.02, respectively), such that ln(1,25(OH)_2_D) = 2.39+0.34(ln(25(OH)D)) for survivors, and 1.88+0.34(ln(25(OH)D)) for non-survivors. For the same level of 25(OH)D, 1,25(OH)_2_D would be lower in non-survivors compared to survivors (p<0.01).

**Figure 4:**
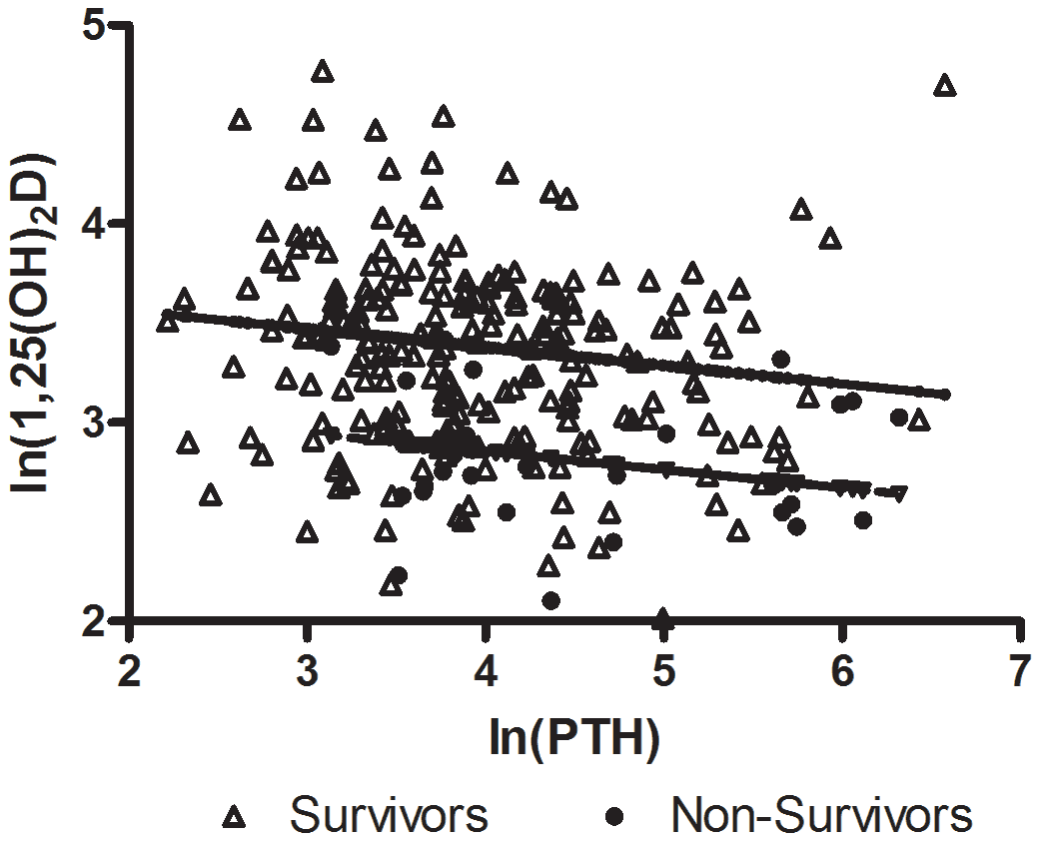
**1,25-dihydroxyvitamin D (1,25(OH)_2_D) as a function of parathyroid hormone (PTH).** The plot shows that in both groups, there is a parallel decrease in ln(1,25(OH)_2_D) for increases in ln(PTH). In the survivors, this relationship is significant (p<0.01). However, the change was not significant in the non-survivors possibly due to small number of subjects (p = 0.80).

Repeated measures regression was utilized to examine the nature of the association between 25(OH)D, 1,25(OH)_2_D, and PTH, which were of physiologic significance in the vitamin D axis. Since these variables and their associations were expected to deviate from assumptions of linear regression, natural log (ln) transformation was applied (Table 4, Figures 3 and 4).

**Table 4:** Lower 1,25-dihydroxyvitamin D (1,25(OH)_2_D) production in non-survivors for the same level of substrate 25-hydroxyvitamin D (25(OH)D) as in survivors.

	25(OH)D (ng/mL)	ln(25(OH)D)	ln(1,25(OH)_2_D)	Derived 1,25(OH)_2_D (pg/mL)
Survivors	18.2	2.90	3.38	29.3
Non-survivors	Same	Same	2.87	17.6

From repeated measures regression analysis (Figure 3), ln(1,25(OH)_2_D) = 2.39+0.34(ln(25(OH)D)) for survivors, and 1.88+0.34(ln(25(OH)D)) for non-survivors. If non-survivors have the same level of substrate 25(OH)D as survivors (e.g. estimated mean of 25(OH)D in Table 2), 1,25(OH)_2_D production will be lower in non-survivors compared to survivors.

All analyses were performed using PASW 18.0 (SPSS, Inc., Chicago, IL, USA) or SAS 9.3 (SAS Institute, Inc., Cary, NC, USA). Statistical significance was defined at p<0.05 (α = 0.05).

## Results

Ninety-one patients were enrolled with average age of 59.1±2.0 years, 52.7% females, and 11.0% deaths at 30 days. At enrollment, there was significant difference in age, stroke comorbidity, APACHE II, and septic shock between survivors and non-survivors (Table 1). At hour 0, 1,25(OH)_2_D and albumin were also significantly different between survivors and non-survivors (Table 2).

### Prognosticators of 30-day Mortality

At baseline (hour 0), all patients met criteria for vitamin D insufficiency (i.e. 25(OH)D<30 ng/mL). There was no significant difference between survivors and non-survivors in 25(OH)D, 24,25(OH)_2_D, and PTH (Table 2). However, 1,25(OH)_2_D was different between the two groups at baseline, 31.5±1.7 vs. 20.4±3.4 pg/mL in survivors vs. nonsurvivors, respectively (p = 0.04).

A univariate analysis including age, stroke comorbidity, APACHE II, septic shock, total calcium, albumin, 25(OH)D, 1,25(OH)_2_D, and PTH was performed for their clinical or statistical association with 30-day mortality (Table 3). The final multivariable model showed that age, total calcium, and 1,25(OH)_2_D were independently associated with 30-day mortality, with odds ratio (95% confidence interval) of 1.08 (1.03,1.12), 0.55 (0.38,0.80), and 0.84 (0.76, 0.92) respectively.

Area under the curve (AUC) of the receiver operating characteristics (ROC) curve for 1,25(OH)_2_D measured at hour 0, 24, 48, and 72 for discriminating 30-day mortality was 0.74, 0.85, 0.89, and 0.87, respectively. The AUC of the ROC curve for total calcium measured at hour 0, 24, 48, and 72 was 0.65, 0.76, 0.83, and 0.81, respectively. Since the AUC for both 1,25(OH)_2_D and total calcium showed the highest AUC’s at hour 48, this time point was chosen for determining the ROC curve of the combined model. The AUC for the ROC curve of the combined model including age, total calcium, and 1,25(OH)_2_D at hour 48 was 0.98 (Figure 1).

Since the model showed 1,25(OH)_2_D to be the best discriminator of mortality (with highest AUC at all time points), the estimated mean 1,25(OH)_2_D over the 72-hour study period was used to determine survival times from study enrollment (hour 0). A cutoff in mean 1,25(OH)_2_D over time of 13.6 pg/mL had the best accuracy (or maximum sum of true positive and true negative) for predicting outcome. Patients with 1,25(OH)_2_D<13.6 pg/mL had 57.1% 30-day survival, compared to 91.7% 30-day survival in patients with 1,25(OH)_2_D level >13.6 pg/mL. The mean survival time for patients with 1,25(OH)_2_D<13.6 pg/mL was 17.6 days, compared to 24.6 days in patients with 1,25(OH)_2_D level >13.6 pg/mL (p<0.01) (Figure 2).

### Effects of Substrate 25(OH)D and PTH Stimulation on 1,25(OH)_2_D

Repeated measures regression analysis of natural log (ln) of 1,25(OH)_2_D as a function of ln(25(OH)D) showed that 1,25(OH)_2_D is positively dependent on 25(OH)D in both survivors and non-survivors (p<0.01 and p = 0.02, respectively) (Figure 3). However, based on the regression equations, ln(1,25(OH)_2_D) = 2.39+0.34(ln(25(OH)D)) for survivors and 1.88+0.34(ln(25(OH)D)) for non-survivors, the same amount of substrate 25(OH)D would result in lower 1,25(OH)_2_D in non-survivors compared to survivors (p<0.01) (Figure 3 and Table 4). Analysis of ln(1,25(OH)_2_D) as a function of ln(PTH) showed that increased levels of PTH did not increase 1,25(OH)_2_D. Instead in both survivors and non-survivors, there was a parallel decrease in 1,25(OH)_2_D with increasing PTH (p<0.01 and p = 0.80, respectively) (Figure 4).

## Discussion

The most salient finding in our study was a demonstration of a decrease in serum 1,25(OH)_2_D in non-survivors compared to the survivors. Not only was the decrement statistically significant, it was also clinically relevant. We found that serum 1,25(OH)_2_D level, and not 25(OH)D, measured over 72 hours was a strong predictor of outcome, with a cutoff of less than or equal to 13.6 pg/mL for determining 30-day mortality in sepsis patients. The implication for the active moiety of vitamin D as an outcome variable could change how we understand and further study the immuno-regulatory mechanisms of sepsis.

The role of vitamin D in sepsis and critically illness has recently received attention since the discovery that Mycobacterium tuberculosis activation of the toll-like receptors (TLR)1/2 pathway further engages a vitamin D-dependent intracellular signaling that results in the upregulation of 1,25(OH)_2_D synthesis, leading to production of LL-37 (also known as cathelicidin) and enhanced monocyte antimicrobial activity [20]. Since the production of 1,25(OH)_2_D is dependent both on the enzyme 1α-hydroxylase and its substrate 25(OH)D, measurement of 25(OH)D level is considered the standard to determine a patient’s overall vitamin D status. Thus, the focus has been determining the association of 25(OH)D with clinical outcome. The majority of studies showed that low levels of 25(OH)D in critical illness were associated with low levels of cathelicidin, increased risk for severe sepsis, hospital length of stay, and mortality [5], [6], [7], [8], [21], [22], [23]. However, contrary to those results, one study showed that 25(OH)D was not a predictor for mortality in sepsis and trauma patients when examined in a multivariable analysis [9]. Consistent with our hypothesis, a few studies have examined 1,25(OH)_2_D levels in cardiac patients and showed that it was associated with poor outcome [12], [24].

To our knowledge, our study is the first to show that 1,25(OH)_2_D, and not 25(OH)D, is strongly associated with 30-day mortality in sepsis. The causal relationship is not known, but what is known is that vitamin D deficiency is associated with increased IL-6, adverse effects on oxidative stress, and increased endothelial adhesion molecule expression, all of which are relevant in the pathogenesis of sepsis [25], [26], [27]. Accordingly, sepsis is associated with a “leaky” vasculature and an increase in cytokine production, abnormalities which are known to be ameliorated by 1,25(OH)_2_D [28]. Moreover, 1,25(OH)_2_D is a major regulator of cathelicidin which act to kill invading microbes [20], [29].

Because of the clinical relevance of our observations, we also examined the potential mechanism of deficiency in this important hormone. The serum level of 1,25(OH)_2_D is determined on one hand, from the conversion of the substrate 25(OH)D to the active moiety 1,25(OH)_2_D by the enzyme 1α-hydroxylase in the kidney, and on the other hand, by its degradation process, the first step of which is the 24-hydroxylation of 1,25(OH)_2_D. The enzyme 25-hydroxyvitamin D 24-hydroxylase controls degradation of both 25(OH)D into 24,25(OH)_2_D, and 1,25(OH)_2_D into 1,24,25(OH)_3_D [30], [31]. Since currently there is no reliable method to measure 1,24,25(OH)_3_D, we assessed the 24-hydroxylase activity by measurement of serum 24,25(OH)_2_D, which was in the normal range and not significantly different in both survivors and non-survivors. This then suggests that 1,25(OH)_2_D degradation was also not different in our two study groups. Thus, a decreased 1,25(OH)_2_D in the non-survivors points to a decreased production, or malfunction of the renal 1α-hydroxylase enzyme, and not due to increased degradation.

We then examined the regulation of the renal 1α-hydroxylase enzyme as a cause of 1,25(OH)_2_D deficiency. There was no difference in the substrate 25(OH)D between survivors and non-survivors at baseline. However, over the 72-hour study period, the estimated mean of 25(OH)D was significantly lower in the non-survivors, and could reduce the amount of 1,25(OH)_2_D produced. Although the substrate was lower in non-survivors, the relationship between substrate 25(OH)D and product 1,25(OH)_2_D (i.e. the slope of ln(1,25(OH)_2_D) as a function of ln(25(OH)D) in our repeated measures regression analysis) was equivalent in both survivors and non-survivors. Based on these relationships we also note that the lower 25(OH)D in non-survivors was insufficient to account for the larger decrease in 1,25(OH)_2_D, such that for the same level of 25(OH)D, there would be a significantly lower 1,25(OH)_2_D in the non-survivors compared to survivors. Our findings suggest that there is some fundamental aberrancy in the renal 1α-hydroxylase enzyme in sepsis, more so in non-survivors. If volume resuscitation during the treatment course of sepsis had impacted vitamin D levels, then we would expect similar decreases in 1,25(OH)_2_D in both survivors and non-survivors based on 25(OH)D levels [32], [33], [34]. There was no difference in the amount of fluids given during the 72-hour study period in survivors compared to non-survivors, 6,024±544 vs. 8,375±870 mL (p = 0.19), but the non-survivors had a 40% lower 1,25(OH)_2_D for the same level of 25(OH)D as in the survivors (Table 4).

Because the circulating level of 1,25(OH)_2_D is generally determined by the kidney, our data suggest that the renal 1α-hydroxylase and the endocrine production of 1,25(OH)_2_D is pivotally disturbed in sepsis. However, certainly as important as the endocrine mechanism, is the autocrine/paracrine function of 1,25(OH)_2_D. This extra-renal action of vitamin D is distributed throughout the body, particularly in white blood cells and epithelial cells, and plays a critical role in innate immunity [35]. Methods are not available to measure the autocrine/paracrine function of 1,25(OH)_2_D in-vivo. However, our finding of a significantly lower circulating level of 1,25(OH)_2_D (when comparing non-survivors to survivors) for the same level of 25(OH)D leads us to speculate that there is an additional abnormality in the autocrine/paracrine function of vitamin D, or extra-renal 1α-hydroxylase activity. If so, this would further compound the total body deficiency of 1,25(OH)_2_D. These observations indicate that there is a subterfuge by sepsis in one of the most important innate immunity defense mechanism for fighting infections.

Our results also suggest an additional aberrancy in the 1α-hydroxylase enzyme. One of the main regulators of renal 1α-hydroxylase activity is serum PTH. PTH was increased in both survivors and non-survivors, but more so in the latter. Generally, an increase in PTH would be accompanied by increased 1,25(OH)_2_D, as seen in hyperparathyroidism [36]. For example, in calcium deficient rats the increase in PTH led to a 12–18 fold increase in serum 1,25(OH)_2_D [37]. Accordingly, we would expect a positive correlation between PTH and 1,25(OH)_2_D in our study. On the contrary, we observed a negative correlation, such that in our repeated measures regression analysis, 1,25(OH)_2_D decreased at high levels of PTH in both survivors and non-survivors. Additionally, for each level of PTH, there appeared to be lower 1,25(OH)_2_D stimulation in non-survivors compared to survivors. These findings strongly suggest a severely dysregulated response of renal 1α-hydroxylase to PTH stimulation. In addition to these changes in vitamin D and PTH we also found that the serum calcium was significantly decreased in the non-survivors compared with the survivors, possibly as a result of the inflammatory response in sepsis [38]. Hypocalcemia may also be due to the impaired PTH action, similar to serum changes in calcium and PTH seen in the 1,25(OH)_2_D null mouse [39]. Taken together, our data suggest a severe disruption in the normal function of calcium regulating hormones in sepsis; and based on our model, this disruption influences, in a large measure, whether patients with sepsis survive.

## Limitations

Our study has several limitations. The study was a prospective study but it was a retrospective analysis which resulted in the data herein. The mortality rate of 11% was appropriate for our patient population with *sepsis* [40]. Our mortality prediction model was based on associations and therefore we cannot conclude that a deficiency of 1,25(OH)_2_D is pathologically causal in sepsis. Due to our small sample size, one could argue that 1,25(OH)_2_D may not be predictive of mortality in a larger sample size. However, we believe the possibility of a Type I error is very low, since the difference in estimated mean 1,25(OH)_2_D in survivors vs. non-survivors was highly significant, p<0.01 (actual p<0.0001).

We analyzed stored plasma samples which would raise the concern regarding the stability of our measurements. Recent studies suggest that multiple freeze-thaw cycles or long term storage up to 24 years does not affect levels of 25(OH)D and its metabolites [41], [42], [43]. However, PTH appears to degrade after 2 months of storage [44], [45]. The fact that we observed significantly high levels of PTH in our study suggests that degradation of PTH from storage would not invalidate our conclusion that the renal 1α-hydroxylase and 1,25(OH)_2_D production are insensitive to PTH stimulation in sepsis. Other authors have similarly described high PTH values in critically ill patients [38], [46]. We also found biological correlation between PTH and 1,25(OH)_2_D, which one would not expect if there had been a random change in the immunoreactivity of PTH in our stored samples. More importantly, a possible underestimation of serum PTH in our study would only further support the conclusion that there is a severe insensitivity of the 1α-hydroxylase to PTH stimulation.

The volume of plasma available for analysis in our study was limited which prohibited additional important measurements such as vitamin D binding protein (DBP), cathelicidin, and cytokine levels. While DBP will influence total levels of 1,25(OH)_2_D, it does not directly affect the biological activity of 1,25(OH)_2_D at local tissues [47]. In fact, 25(OH)D and 1,25(OH)_2_D have increased ability to induce monocyte production of the antimicrobial protein cathelicidin in DBP knockout mouse [48]. Thus not having DBP measurements in our study does not limit our observation that decreased 1,25(OH)_2_D in sepsis patients is associated with mortality. Furthermore, even if DBP was low in our patient population, the observed high PTH levels would suggest that the biological activity of unbound free 1,25(OH)_2_D was low, and associated with the observed poor outcome.

### Conclusion

Our study showed that 1,25(OH)_2_D is a novel prognosticator of outcome in sepsis. This naturally leads to the question of its potential as a therapeutic target. While animal sepsis models examining 1,25(OH)_2_D administration have shown promise, there is no human randomized clinical trial [49], [50], [51]. However, we are cautious with its potential as the “magic bullet” in the treatment of sepsis due to the complexity of the vitamin D axis. Future studies will also need to elucidate the immuno-regulatory mechanisms of vitamin D in relation to the inflammatory cascade of sepsis that has been much studied in the past decades.

In designing further studies, our findings must be taken into consideration. Deficiency in 1,25(OH)_2_D may be due to 1) a failure of endocrine 1α-hydroxylation in the kidney to the stimulatory actions of PTH, and 2) an impairment of the production of 1,25(OH)_2_D by immune cells. Providing vitamin D as a therapy, and targeting 25(OH)D levels alone may not necessarily be totally effective. To correct the abnormalities in vitamin D metabolism, we would need to correct both serum 25(OH)D and 1,25(OH)_2_D levels by the administration of vitamin D and 1,25(OH)_2_D, respectively.
